# Supplementary material for: Staged developmental mapping and X chromosome transcriptional dynamics during mouse spermatogenesis
Source: Nat Commun. 2019 Mar 19;10:1251. doi: 10.1038/s41467-019-09182-1 (PMC6424977; doi:10.1038/s41467-019-09182-1)
Supplement: Supplementary file 1 — Supplementary Information [file 41467_2019_9182_MOESM1_ESM.pdf]

Staged developmental mapping and X chromosome transcriptional dynamics during mouse spermatogenesis

Ernst et al.

SUPPLEMENTARY FIGURES

Supplementary Fig. 1. Histological examples of different tubule stages and sub-stages of cell-types. **(p.S3)**.

Supplementary Fig. 2. Batch- and sample effects across all scRNA-Seq samples. **(p.S4)**.

Supplementary Fig. 3. Cell-type composition in juvenile samples during the first wave of spermatogenesis. **(p.S5)**.

Supplementary Fig. 4. Marker genes and transcriptional changes during somatic cell-type differentiation. **(p. S7)**.

Supplementary Fig. 5. Spermatogonial sub-populations at P5, P10 and P15. **(p.S9)**.

Supplementary Fig. 6. Detection of cell-types with low transcriptional complexity at different timepoints during spermatogenesis. (**p.S10**).

Supplementary Fig. 7. The presence of the human chromosome 21 has minimal impact on the mouse genome's gene expression. (**p.S12**).

Supplementary Fig. 8. Dynamic expression of histone variants and canonical histones during spermatogenesis. (**p.S14**).

Supplementary Fig. 9. X chromosome inactivation during meiosis and expression of X-linked multi-copy gene families during spermatogenesis. (**p.S15**).

Supplementary Fig. 10. CUT&RUN profiling of histone mark dynamics during spermatogenesis. (**p.S17**).

Supplementary Fig. 11. Enrichment of repeat elements in regions of high H3K9me3 signal. (**p.S19**).

Supplementary Fig. 12. Epigenetic regulation of spermatid-specific and non-specific genes. (**p.S20**).

.

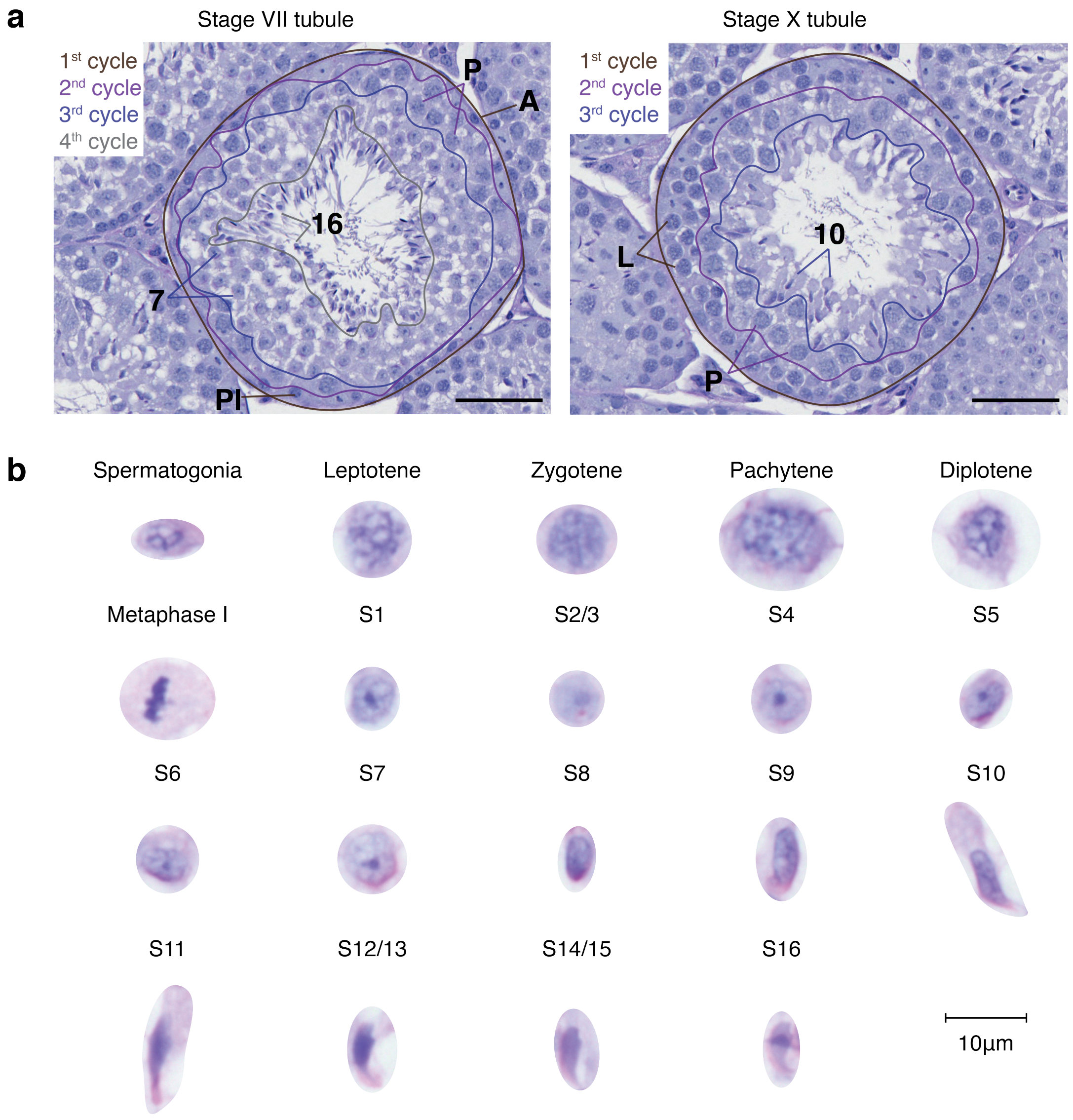

Supplementary Fig. 1. Histological examples of different tubule stages and sub-stages of cell-types.

(a) Higher magnification of two tubules depicted in Fig. 1A. The PAS-stained cross-sections depict tubules in Stage VII and Stage X, with the different cell layers indicated by coloured lines. Stage VII tubules contain 4 different layers with germ cells from different generations that are approximately 8.6 days apart, whereas Stage X tubules only contain three layers. Each layer contains a certain set of germ cells depending on the epithelial stage (see **Fig. 1b**). Stage VII contains preleptotene spermatocytes (Pl) in the first layer, pachytene spermatocytes (P) in the second layer, S7 round spermatids (7) in the third layer and S16 elongating spermatids in the fourth innermost layer. Stage X tubules contain leptotene spermatocytes (L) in the first layer, pachytene spermatocytes (P) in the second layer and only one generation of round-to-elongating spermatids at S10 (10) in the innermost layer. Original magnification 40X.

(b) High resolution images of PAS-stained germ cells depicting the different nuclear morphology of spermatocytes and spermatids as well as acrosome development in spermatids. Original magnification 60X; scale bar represents 10 μm.

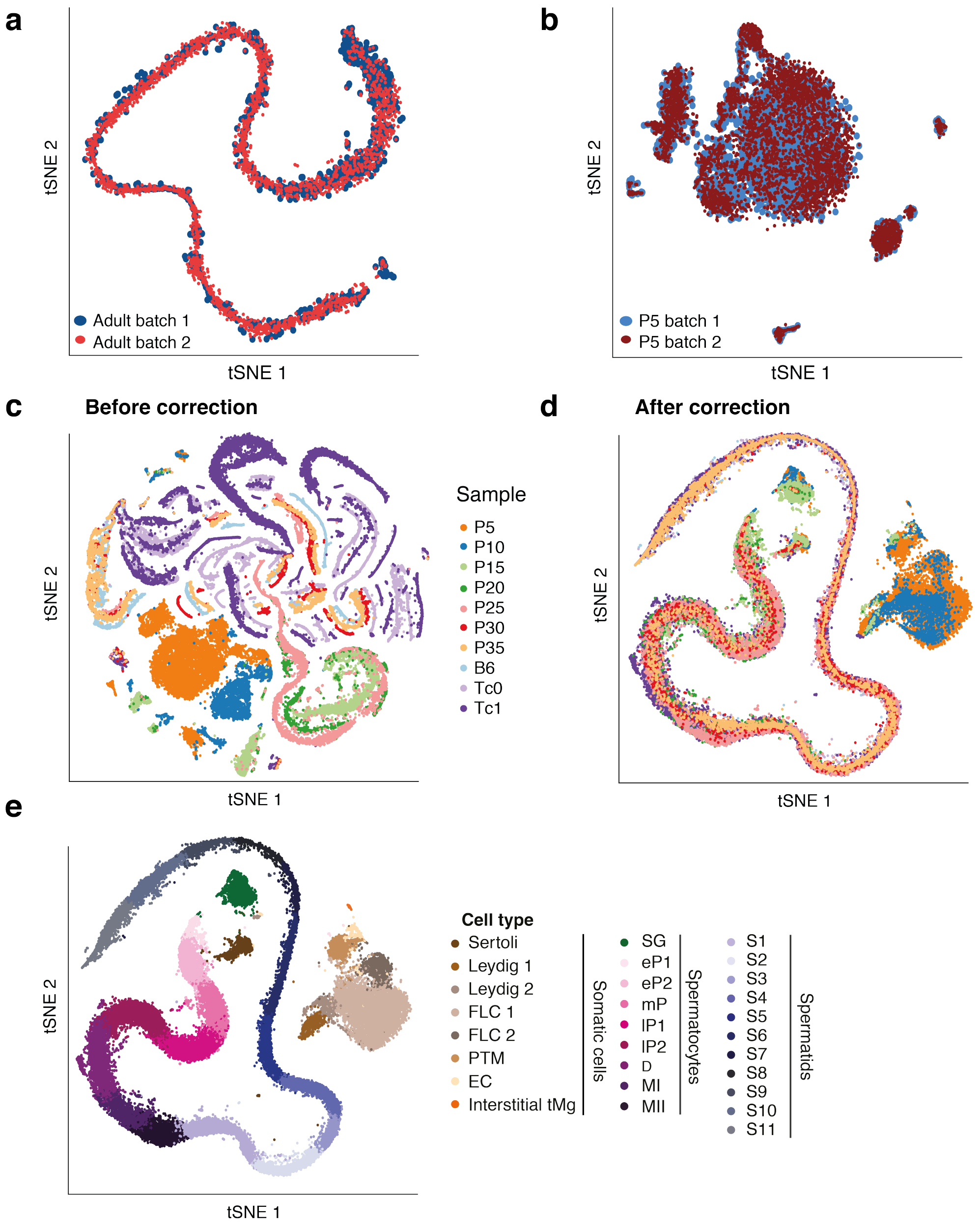

Supplementary Fig. 2. Batch- and sample- effects across all scRNA-Seq samples.

(a) and **(b)** Low dimensionality representation (tSNE) of CellRanger quality filtered cells (**Methods**) from 2 replicates of adult B6 animals **(a)** and 2 replicates of P5 animals **(b)**. The tSNE was calculated on $\log_2$-transformed normalised counts.

(c) and **(d)** tSNE representation of $\log_2$-transformed normalised counts **(c)** and batch-corrected counts **(d)** across all samples (**Methods**). Cells are coloured based on the biological time-point they were sampled from (see **Supplementary Data 1**).

(e) tSNE representation of batch-corrected cells as in **(d)**. Cells are labelled based on their cell-type identity as obtained by clustering and cell-type annotation (**Methods**). FLC – Fetal Leydig Cells, PTM – Peritubular Myoid Cells, EC – Endothelial Cells, tMg – testicular macrophages, SG – Spermatogonia, eP - early-pachytene spermatocytes (SC), mP – mid-pachytene SC, lP – late-pachytene SC, D – diplotene SC, MI – metaphase I, MII – metaphase II, S1-11 – step 1-11 spermatids.

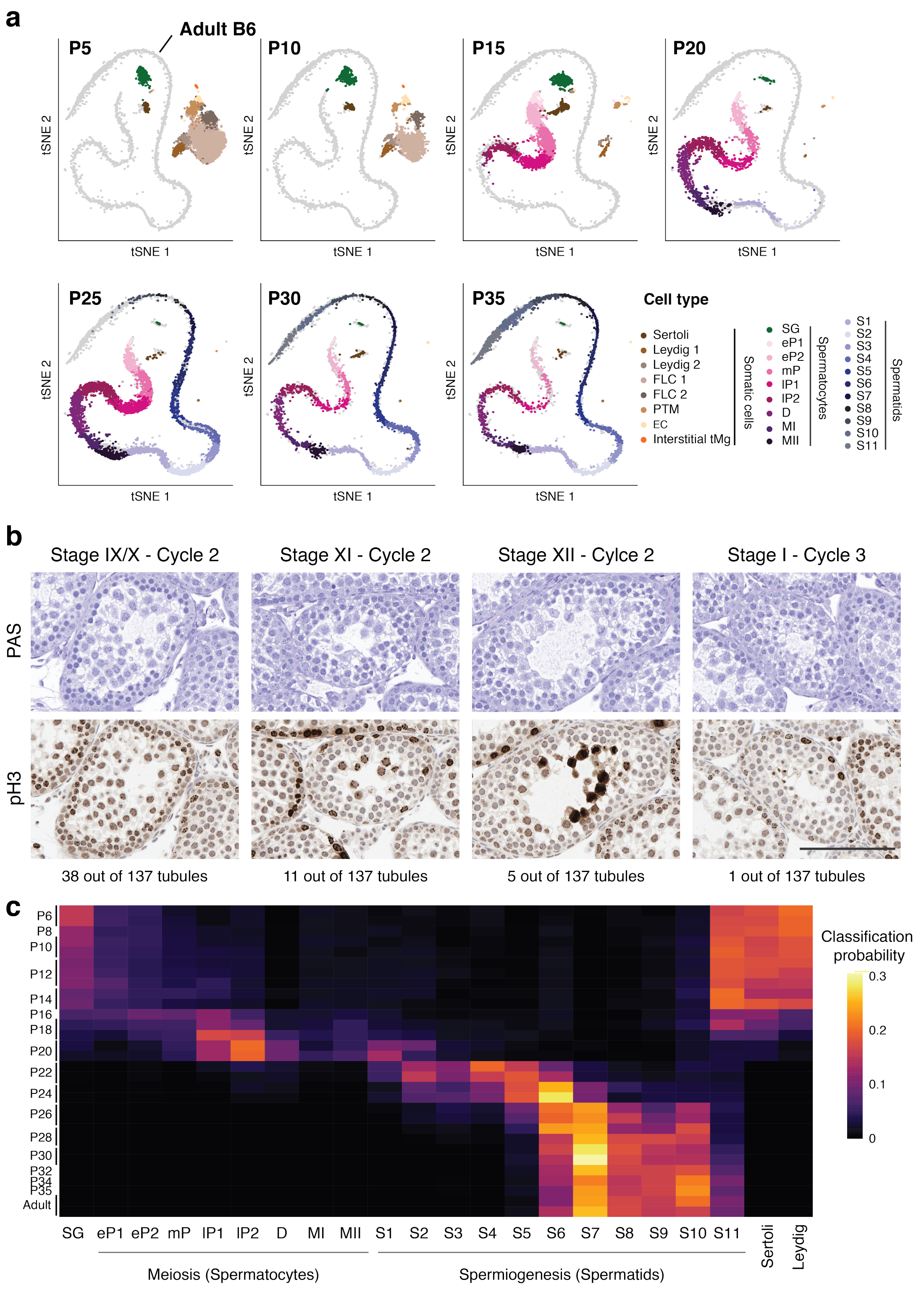

Supplementary Fig. 3. Cell-type composition in juvenile samples during the first wave of spermatogenesis.

(**a**) tSNE representation of juvenile cells that were mapped to cells isolated from adult mice. Grey dots indicate all cells from adult animals that were used as a reference for cell mapping (**Methods**). Coloured dots represent cells isolated at each sampled stage during the first wave of spermatogenesis. Clustering was performed across all cells after cell mapping (**Methods**). FLC – Fetal Leydig Cells, PTM – Peritubular

Myoid Cells, EC – Endothelial Cells, tMg – testicular macrophages, SG – Spermatogonia, eP - early-pachytene spermatocytes (SC), mP – mid-pachytene SC, lP – late-pachytene SC, D – diplotene SC, MI – metaphase I, MII – metaphase II, S1-11 – step 1-11 spermatids.

(b) Representative images of tissue sections from P20 testes stained with Periodic-Acid Schiff (PAS) and Immunohistochemistry (IHC) against phospho-Histone H3 (pH3) depicting tubules in late stages of the second epithelial cycle (Stages IX-XII) and Stage I of the third epithelial cycle. pH3 signal across entire tissue cross-section was quantified and staining intensity in spermatocytes was used to stage tubules. Scale bar represents 100 μm; original magnification 40X.

(c) Probabilistic mapping of bulk RNA-Seq libraries to the cell clusters identified in the adult scRNA-Seq data using a random forest approach (**Methods**). The colour gradient indicates the probability with which a bulk sample can be assigned to the specific cell cluster.

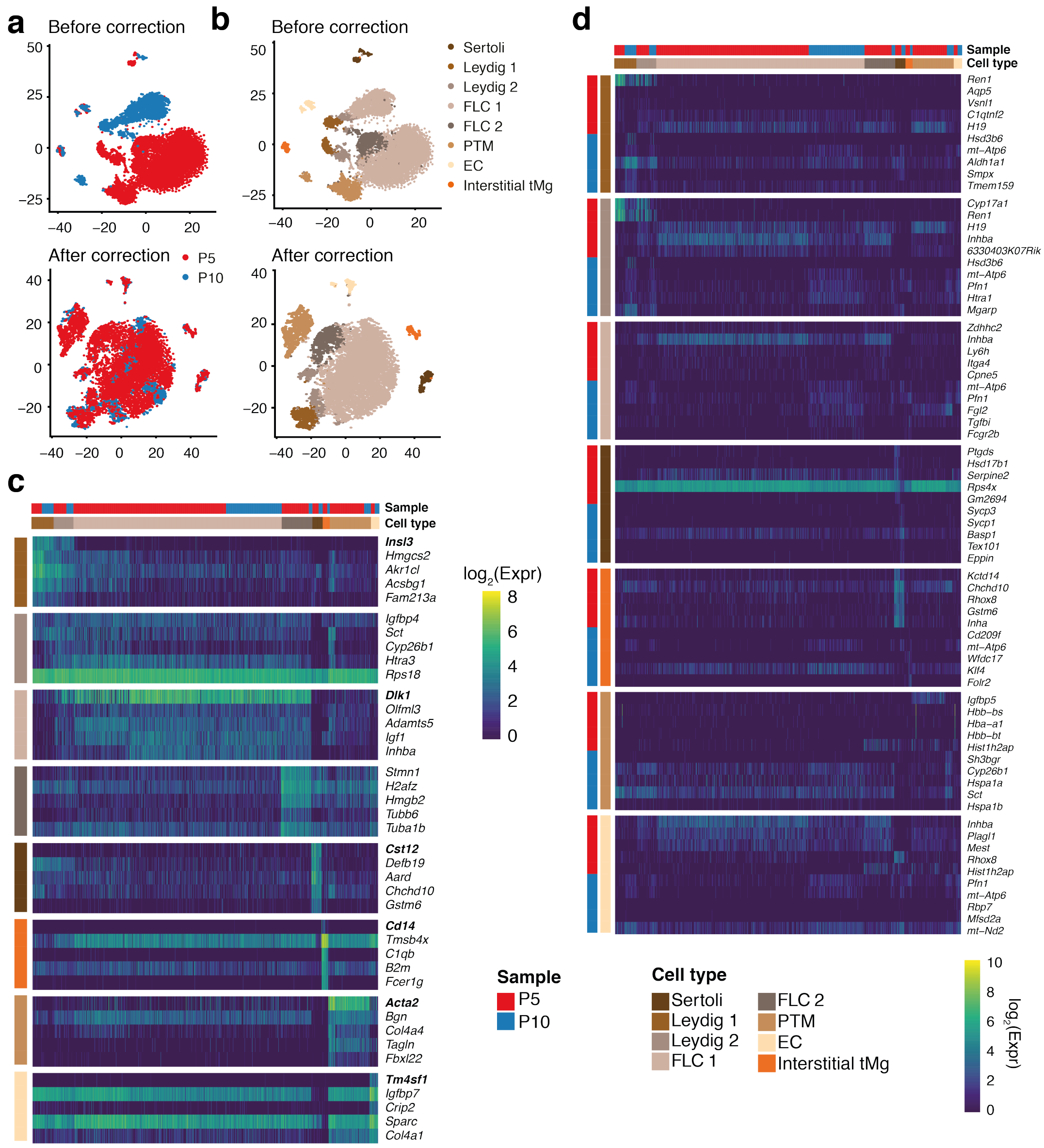

Supplementary Fig. 4. Marker genes and transcriptional changes during somatic cell-type differentiation.

(a)-(b) tSNE representation of somatic cell-types from P5 and P10 animals before (upper) and after (lower) batch correction. Cells in **(a)** are labelled based on the time-point at which they were captured, cells in **(b)** are labelled based on their cell-type as identified by unbiased clustering (**Methods**). FLC – Fetal Leydig Cells, PTM – Peritubular Myoid Cells, EC – Endothelial Cells, tMg – testicular marcrophages.

(c) Heatmap representation of cell-type-specific marker genes for all somatic cell-types across P5 and P10 samples (**Methods**). Bolded genes indicate previously described markers for the following cell-types: Leydig cells (*Insl3*), Fetal Leydig cells (*Dlk1*), Sertoli cells (*Cst12*), testicular macrophages (*Cd14*), peritubular myoid cells (*Tm4sf1*), endothelial cells (*Acta2*). For each cell-type, one annotated marker gene and the top 4 marker genes are displayed. Cells are ordered based on their cell-type identity and sample.

(d) Heatmap representation of genes that were detected to be differentially expressed (DE) between P5 and P10 for each somatic cell-type. The top 5 DE genes

for each time-point within each somatic cell-type are displayed. Genes were ordered based on the cell-type and sample (colour labelling in left column). Cells are ordered based on their cell-type identity and sample.

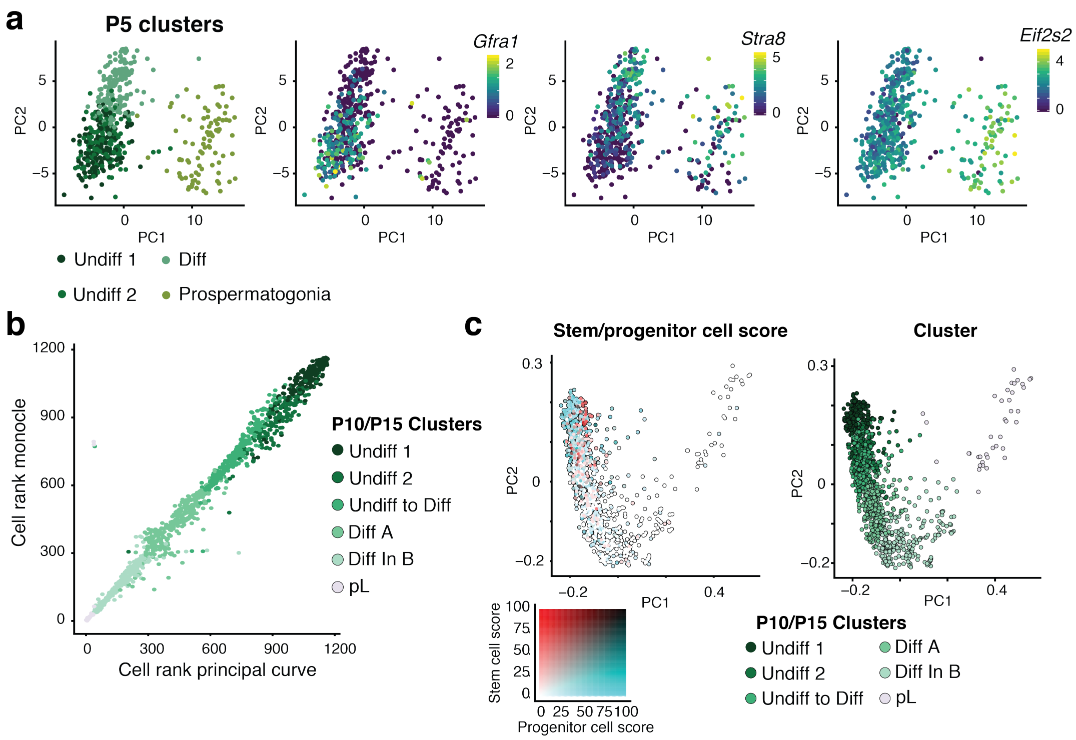

Supplementary Fig. 5. Spermatogonial sub-populations at P5, P10 and P15.

(a) PCA representation of P5 germ cells. The first panel depicts cluster annotation of cells. The remaining panels display the log_2-transformed, normalised expression of cluster-specific marker genes (*Gfra1*, *Stra8*, and *Eif2s2*) (**Supplementary Data 5**).

(b) Batch-corrected transcriptomes of P10 and P15 germ cells were ordered along their differentiation trajectory using a principal curve regression or monocle (**Methods**). The x-axis represents each cell's rank as estimated by the principal curve approach while the y-axis represents each cell's rank using monocle. Cells were labelled based on their cluster annotation (see **Fig. 3b**).

(c) For each spermatogonia of the P10 and P15 samples, the fraction of stem cell markers (stem cell score) and the fraction of progenitor cell markers (progenitor cell score) were computed (**Methods**) (La et al., 2018). Cells were coloured based on their stem and progenitor cell score. The right panel shows the germ cell cluster annotation as in **Fig. 3b**.

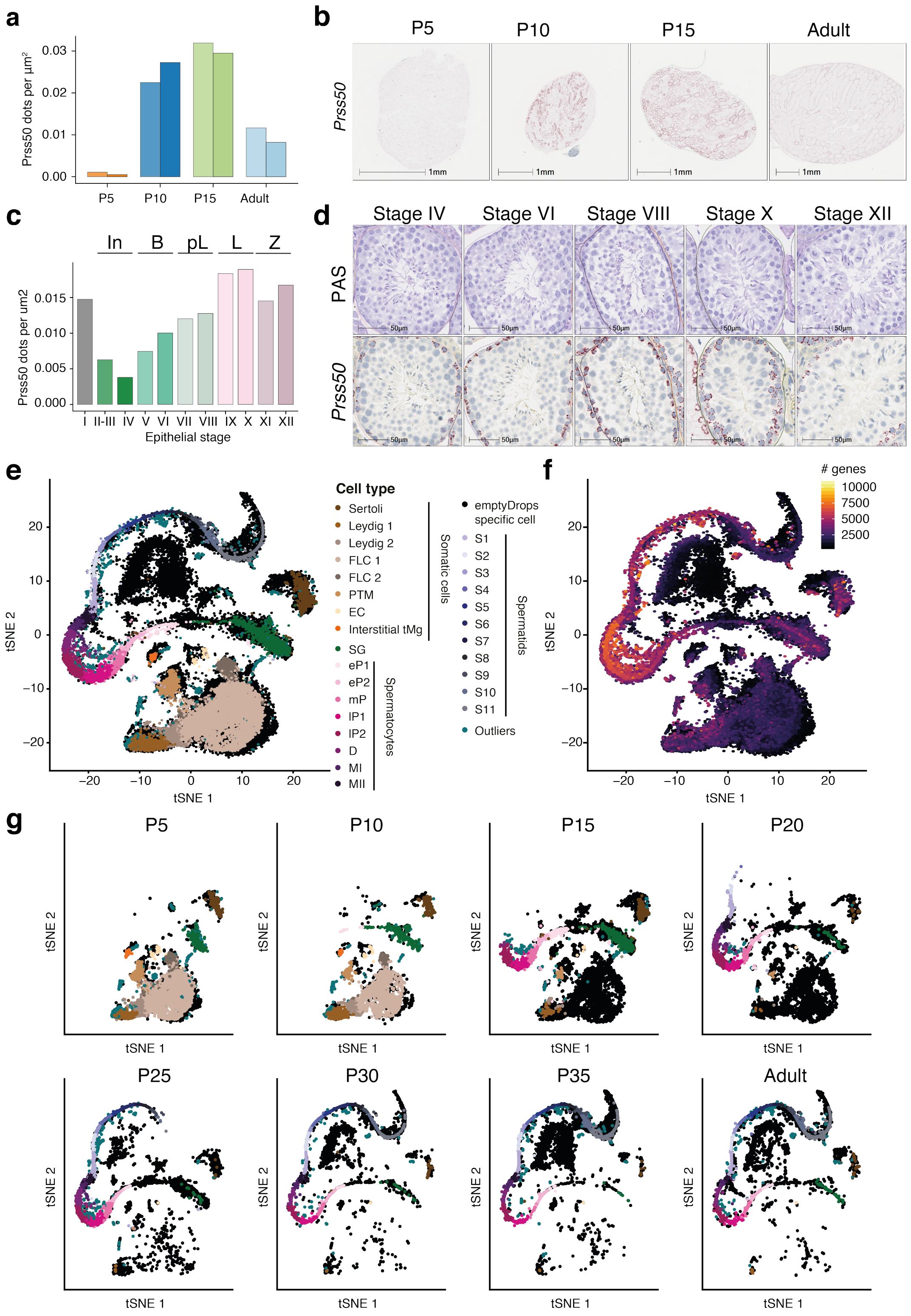

Supplementary Fig. 6. Detection of cell-types with low transcriptional complexity at different timepoints during spermatogenesis.

(a) Quantification of RNAScope dots for *Prss50* per μm^2 across entire tissue cross-section from P5, P10, P15, and adult animals (n = 2). Source data are provided as a Source Data file.

(b) Representative images of tissue cross-sections from different developmental time-points stained with RNA ISH for *Prss50*. Original magnification was 2X; scale

bars represent 1 mm.

(c) Quantification of RNAScope dots for *Prss50* per μm^2 within tubules at different epithelial stages. A total of 160 tubules was quantified across entire tissue cross-section from adult B6 animal. Labels above the bars display which sub-type of spermatogonia are present at the different epithelial stages (see **Fig. 1b**). Source data are provided as a Source Data file.

(d) Representative images of seminiferous tubules at different epithelial stages stained with PAS or RNA ISH for *Prss50* using RNAScope on tissue sections from adult B6 animals. Original magnification was 40X, scale bars represent 50 μm.

(e) tSNE representation of cells selected by the EmptyDrops filtering strategy (**Methods**). Coloured dots represent annotated cell-types detected using the default CellRanger filtering pipeline while black dots represent cells newly detected by the emptyDrops filtering. CellRanger selected cells that were removed from downstream analysis are labelled as “Outliers”. FLC – Fetal Leydig Cells, PTM – Peritubular Myoid Cells, EC – Endothelial Cells, tMg – testicular macrophages, SG – Spermatogonia, eP - early-pachytene spermatocytes (SC), mP – mid-pachytene SC, lP – late-pachytene SC, D – diplotene SC, MI – metaphase I, MII – metaphase II, S1-11 – step 1-11 spermatids.

(f) Visualisation of the number of genes expressed (> 0 counts) per cell across all EmptyDrops selected cells.

(g) tSNE representation of EmptyDrops filtered cells for each of the sampled time-points. Colouring corresponds to (**e**).

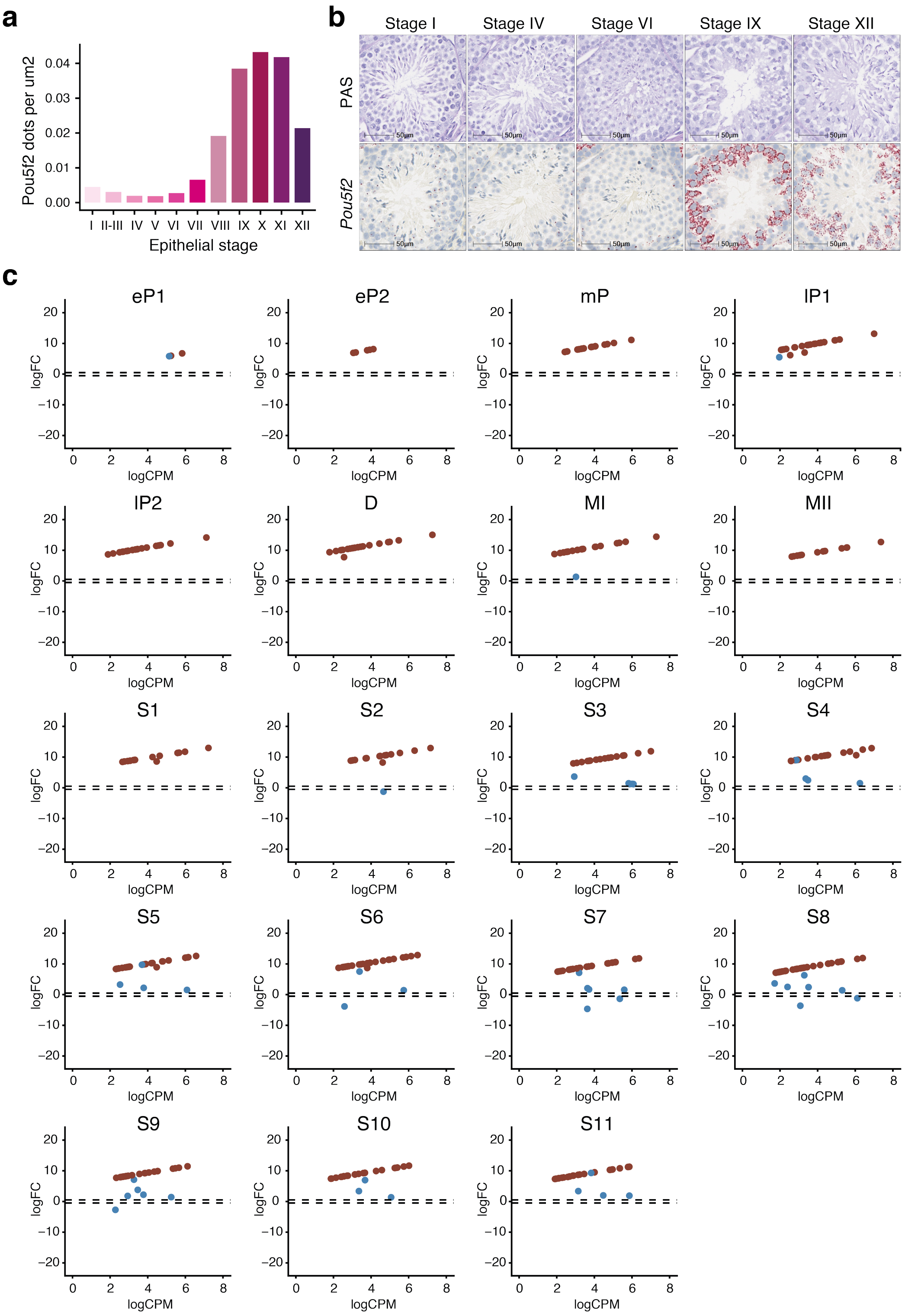

Supplementary Fig. 7. The presence of the human chromosome 21 has minimal impact on the mouse genome's gene expression.

(a) Quantification of RNAScope dots for *Pou5f2* per μm^2 within tubules at different epithelial stages. A total of 217 tubules was quantified across entire tissue cross-section from adult B6 animal. Source data are provided as a Source Data file.

(b) Representative images of seminiferous tubules at different epithelial stages stained with PAS or RNA ISH for *Pou5f2* using RNAScope on tissue sections from

adult B6 animals. Original magnification was 40X, scale bars represent 50 μm.

(c) Differential expression was tested between 3 replicates of Tc0 animals and 4 replicates of Tc1 animals within each cell-type (after removing lowly expressed genes, averaged log_2-transformed normalised expression > 0.1). For genes with statistically significant change in mean expression (absolute log_2-fold change > 0.5, FDR < 0.1), the log_2-fold change in expression (logFC, Tc1/Tc0) was plotted against the log_2-transformed counts per million (logCPM). Differentially expressed genes from human chromosome 21 are labelled as red dots while differentially expressed genes from the mouse genome are labelled as blue dots. The dashed lines show a logFC = 0.5 and logFC = -0.5.

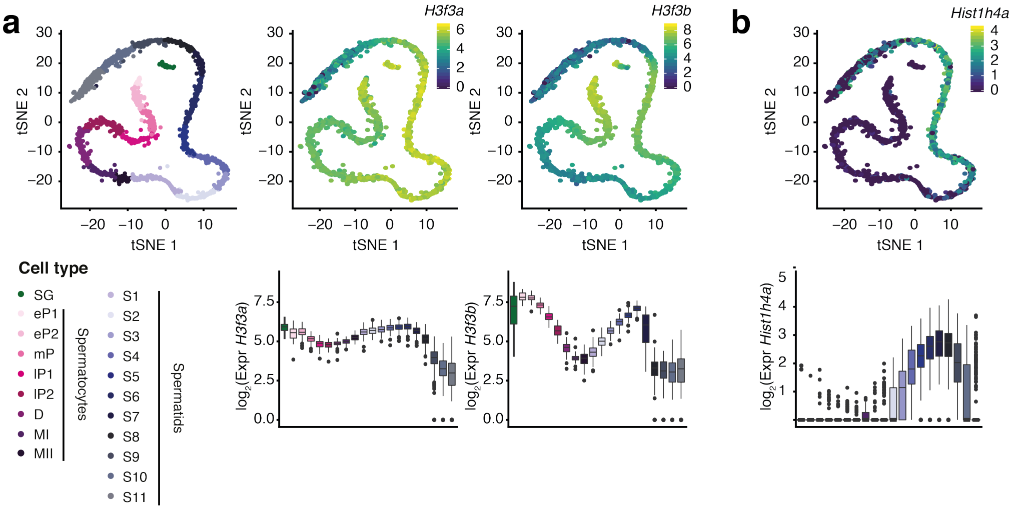

Supplementary Fig. 8. Dynamic expression of histone variants and canonical histones during spermatogenesis.

(a) Expression of *H3f3a* (middle panel) and *H3f3b* (right panel) across the different germ cell populations. The upper panels show a tSNE representation of gene expression where the colour scale represents the log_2-transformed, normalised counts. The lower panels show the expression of these genes in form of boxplots.

(b) Similar visualisation as in **(a)** of *Hist1h4a* expression across germ cells.

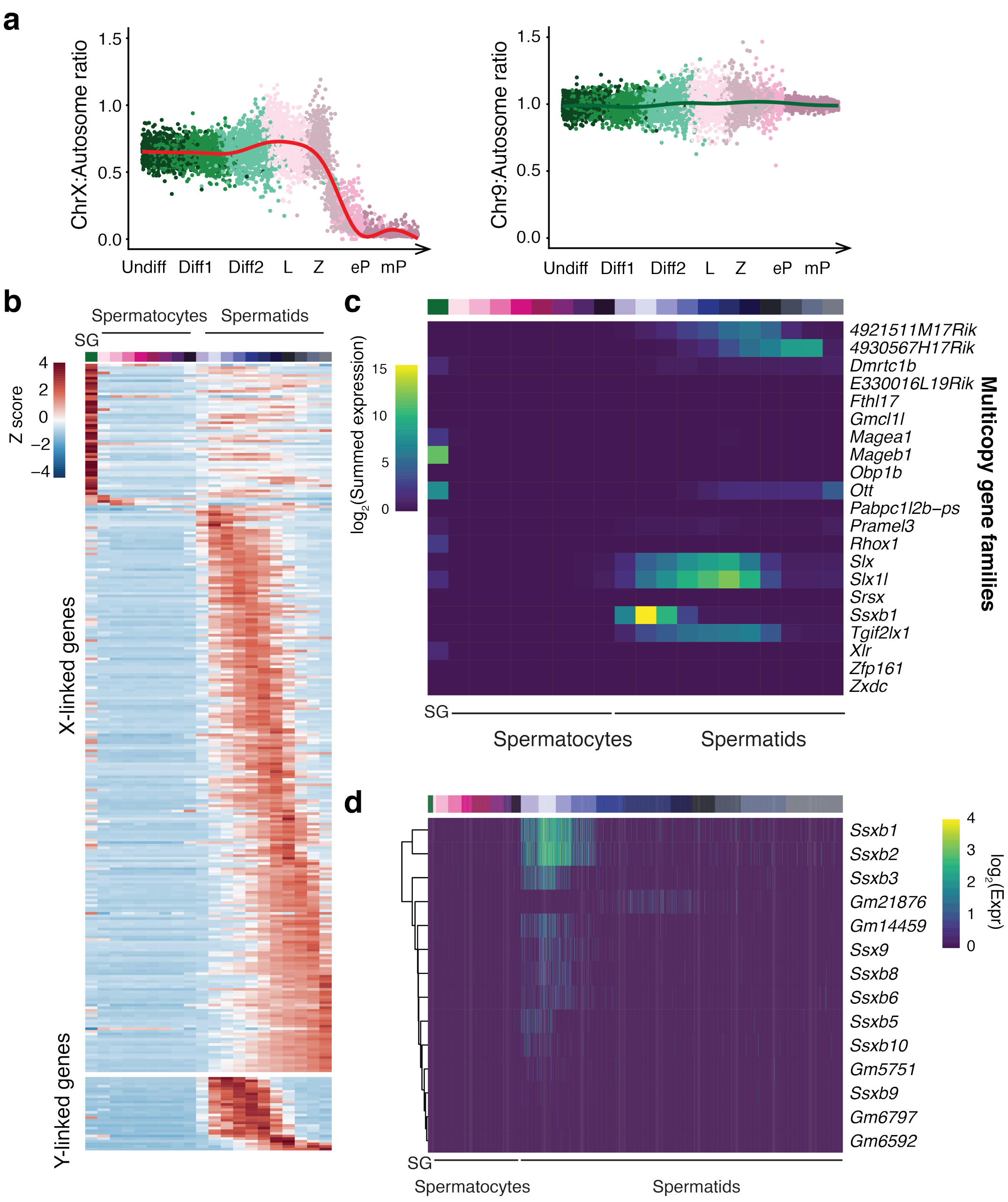

Supplementary Fig. 9. Sex chromosome inactivation during meiosis and expression of X-linked multi-copy gene families during spermatogenesis.

(a) For each EmptyDrops filtered germ cell of the P15 sample, the ratio of mean expression of genes on ChrX (left) or Chr9 (right) to the mean expression of genes across all autosomes was calculated (**Methods**). Cells were ordered along their differentiation time-course using principal curve regression. Undiff – Undifferentiated spermatogonia, Diff – Differentiating spermatogonia, L – Leptotene spermatocytes (SC), Z – Zygotene SC, eP – early-pachytene SC, mP – mid-pachytene SC.

(b) Log_2-transformed normalised gene expression of all X chromosomal (top) and Y chromosomal (bottom) genes (> 0.1 averaged log_2-transformed counts) was averaged within each cell-type. The Z score was computed across all cells. Genes were ordered based on their peak expression.

(c) Log_2-transformed normalised reads for genes with high sequence similarity (> 90%) were summed across the multi-copy gene family (**Methods**). Summed counts

were averaged within the different cell-types. SG - spermatogonia.

(d) Expression of all genes contained in the *Ssxb* gene family. Cells were ordered based on their cell-type labels. Genes were clustered based on the Euclidean distance between their log_2-transformed normalised expression profiles. SG - spermatogonia.

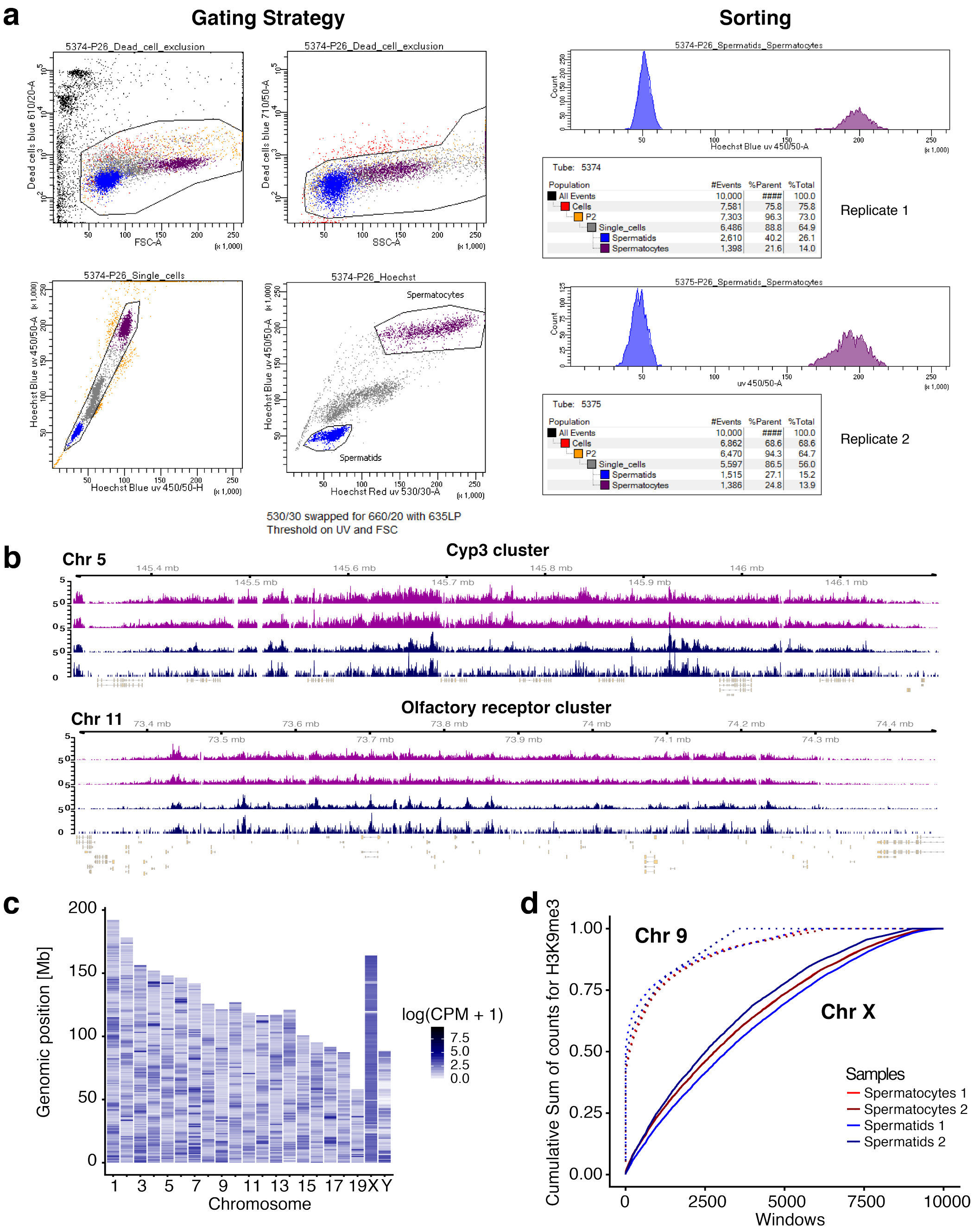

Supplementary Fig. 10. CUT&RUN profiling of histone mark dynamics during spermatogenesis.

(a) Left-hand side depicts scatter plots representing the gating strategy for the isolation of spermatocytes and spermatids. (Top Left) Dead cell exclusion based on propidium iodide (PI) (Dead cells blue 610/20-A) versus forward scatter (FSC-A); (Top Right) Dead cell exclusion based on PI (Dead cells blue 710/50-A) versus side scatter (SSC-A); (Bottom Left) removal of doublets based on Hoechst Blue versus Hoechst Blue (Hoechst Blue uv 450/50-A); (Bottom Right) identification of spermatogenic cell populations based on Hoechst Blue versus Hoechst Red (Hoechst Red uv 530/30 in axis label, but 660/20 with 635 longpass (LP) filter was used). Threshold was set on UV and FSC. Right-hand side shows the counts per cell-type and their proportions within the parent cell populations for two biological

replicates at P26.

(b) H3K9me3 signal across tissue-specific gene clusters on autosomes depicting a cytochrome P450 gene cluster on chromosome 5 and an olfactory receptor gene cluster on chromosome 11. Two replicates for spermatocytes (upper tracks) and spermatids (lower tracks) from P26 animals are shown.

(c) Whole chromosome view of H3K9me3 signal in 20Kb bins depicting one replicate of spermatocytes from P26 animal. The log-transformed scale normalised counts (counts per million + 1) are displayed per bin. The y-axis indicates the position (in megabases, Mb) of each bin on the chromosome.

(d) Cumulative summed counts per million across 10,000 randomly sampled windows (1,000 bp width) visualising the distribution of the H3K9me3 signal across chromosome 9 (dashed line) and chromosome X (solid line).

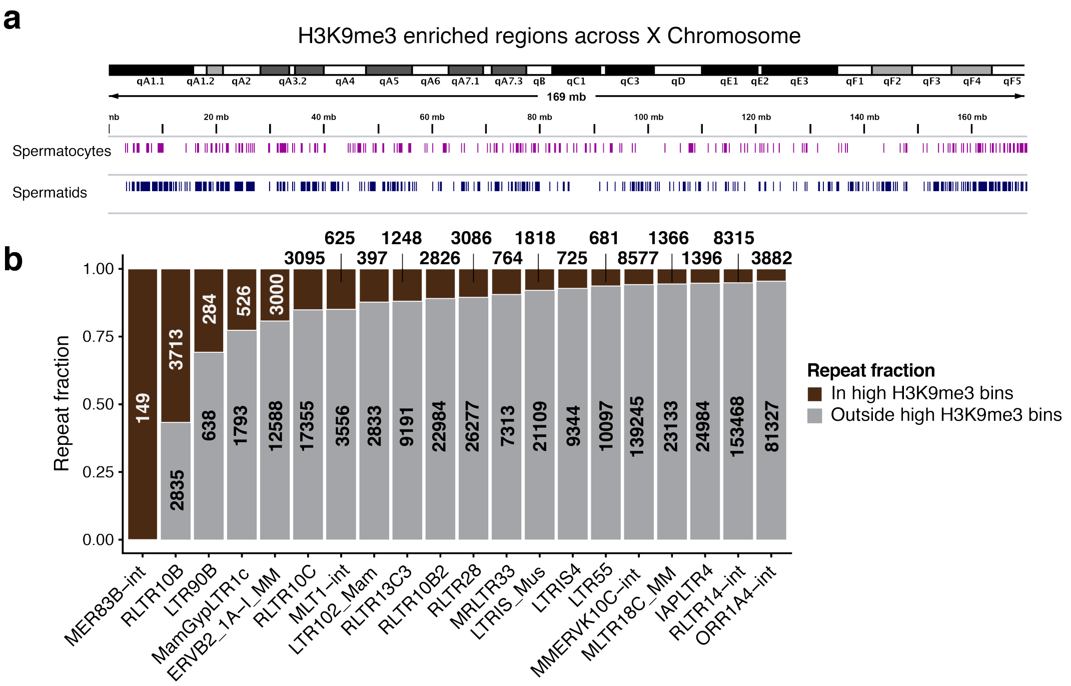

Supplementary Fig. 11. Enrichment of repeat elements in regions of high H3K9me3 signal on the X chromosome.

(a) The top 1,000 windows with highest H3K9me3 signal on the X chromosome (1,000 bp width, CPM) were merged using a tolerance of 1,500 bp (**Methods**). Representative tracks of one replicate in spermatocytes (upper track) and one replicate in spermatids (lower track) are shown from one P26 animal.

(b) The enrichment of repeat elements within the regions of high H3K9me3 was tested compared to the rest of the X chromosome (**Methods**) using a Fisher's Exact test. The fraction of bases for each repeat family within (brown) and outside (grey) the regions of high H3K9me3 signal are displayed, showing the top 20 hits. Additionally, the number of bases within and outside the regions with highest H3K9me3 signal are shown.

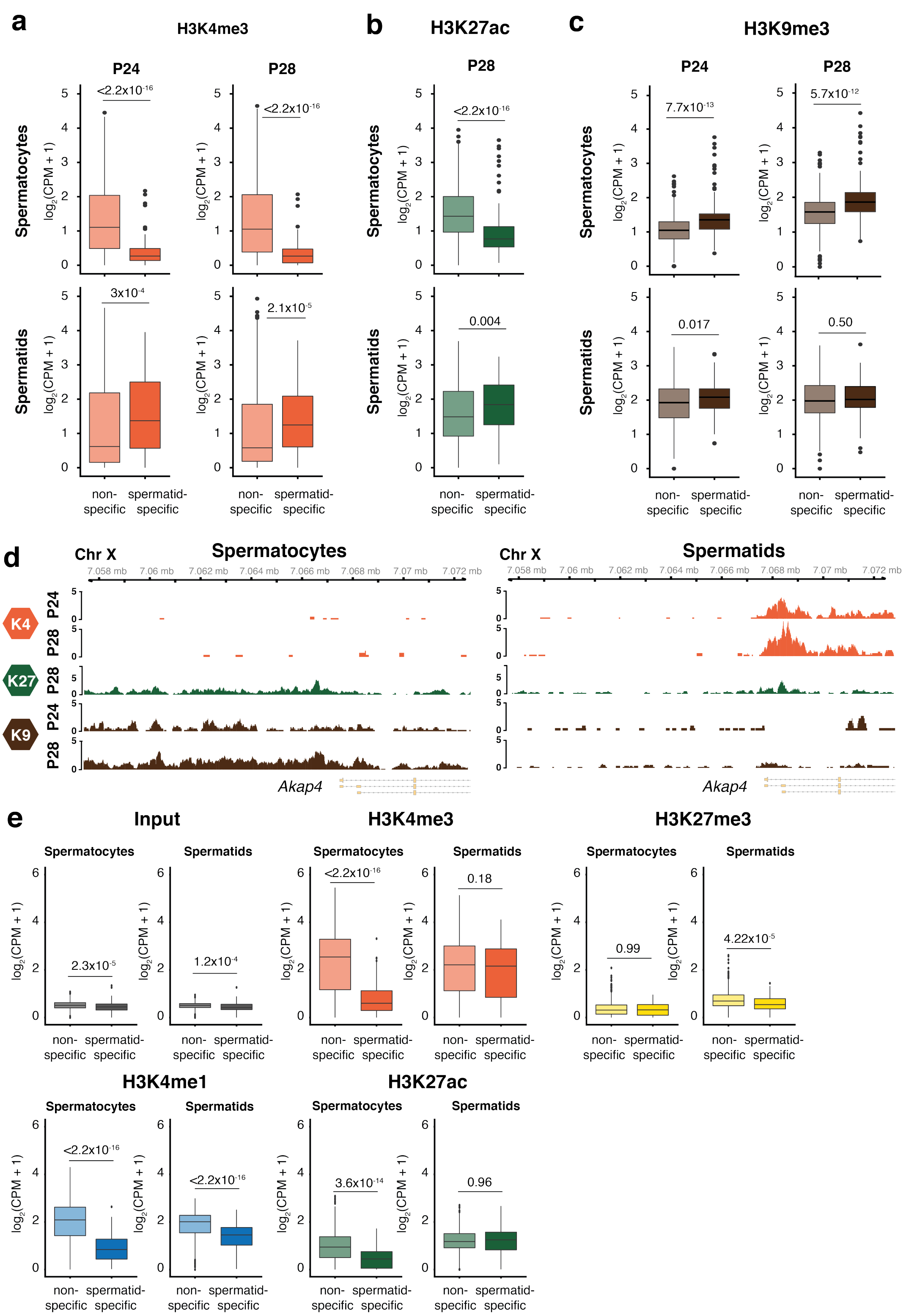

Supplementary Fig. 12. Epigenetic regulation of spermatid-specific and non-specific genes on the X chromosome.

(a) - **(c)** CUT&RUN samples for additional developmental time-points: P24 and P28 for H3K4me3 and H3K9me3 as well as P28 for H3K27ac. Boxplots of H3K4me3 **(a)**, H3K27ac **(b)** and H3K9me3 **(c)** display Counts Per Million (CPM) in promoter regions of spermatid specific (n=128) and non-spermatid specific (n=622) genes for spermatocytes (top) and spermatids (bottom). Counts were averaged across two

biological replicates. Statistical significance when testing for differences in histone mark abundance is displayed in form of p-values using a Wilcoxon-Mann-Whitney test. X-linked spermatid-specific and non-specific genes were defined in **Fig. 7c**.

(d) Genome tracks of H3K4me3, H3K27ac and H3K9me3 for *Akap4* in Spermatocytes (left) and Spermatids (right) at different developmental time-points. Reads were scaled by library size.

(e) ChIP-Seq data of H3K4me3, H3K27me3, H3K4me1 and H3K27ac from *Hammoud et al.* (2014) was analysed for signal enrichment in promoters of spermatid-specific and non-specific genes (**Methods**). The input control was visualised to highlight even capture of reads in both promoter categories. Statistical significance when testing for differences in histone mark abundance is displayed in form of p-values using a Wilcoxon-Mann-Whitney test.

REFERENCES

Hammoud, S.S., Low, D.H.P., Yi, C., Carrell, D.T., Guccione, E., and Cairns, B.R. (2014). Chromatin and Transcription Transitions of Mammalian Adult Germline Stem Cells and Spermatogenesis. Cell Stem Cell *15*, 239–253.

La, H.M., Mäkelä, J.-A., Chan, A.-L., Rossello, F.J., Nefzger, C.M., Legrand, J.M.D., Seram, M.D., Polo, J.M., and Hobbs, R.M. (2018). Identification of dynamic undifferentiated cell states within the male germline. Nat. Commun. *9*, 2819.
