# Supplementary material for: Staged developmental mapping and X chromosome transcriptional dynamics during mouse spermatogenesis
Source: Nat Commun. 2019 Mar 19;10:1251. doi: 10.1038/s41467-019-09182-1 (PMC6424977; doi:10.1038/s41467-019-09182-1)
Supplement: Supplementary file 13 — Description of Additional Supplementary Files [file 41467_2019_9182_MOESM13_ESM.pdf]

Supplementary Data legends

Title: Supplementary Data 1: **Overview and quality metrics of scRNA-Seq, bulk RNA-Seq and CUT&RUN libraries.**
Description: 1st sheet: Quality metrics of droplet-based scRNA-Seq. Sample: Stage and genotype information for all samples. Library: sample identifier. CellRanger filter: Number of retained cells after default thresholding using the CellRanger *counts* function. After quality control: Number of cells obtained after quality control. Assigned Cell-type: Number of cells that fall into annotated clusters (removing outlying cells). EmptyDrops filter: Number of cells retained after using the EmptyDrops function controlling the FDR to 1%. EmptyDrops quality control: Number of cells obtained after quality control of the EmptyDrops filtered cells.
2nd sheet: Animal age (in days) information of bulk RNA-Seq libraries sampled throughout the first wave of spermatogenesis.
3rd sheet: Age and chromatin mark information of CUT&RUN libraries.

Title: Supplementary Data 2: **Cluster-specific marker genes for all cell clusters identified in adult B6 animals.**
Description: Marker genes were detected across multiple pairwise comparisons using the findMarker function implemented in scran (**Methods**). The sheet name indicates the cell-type for which marker genes are listed. Genes are ranked based on their top rank across all pairwise comparisons. Top: the minimum rank across all pairwise comparisons. Columns labelled with “logFC.X”: $\log_2$-fold change between the current cell-type and the cell-type X. GeneName: Gene symbol for marker genes. SG - Spermatogonia, eP – early-pachytene spermatocytes (SC), mP – mid-pachytene SC, lP – late-pachytene SC, D – Diplotene SC, MI – Metaphase I, MII – Metaphase II, S1-11 – Step 1-11 Spermatids.

Title: Supplementary Data 3: **Cluster-specific marker genes for somatic cell-types in P5 and P10.**
Description: Marker genes were detected across multiple pairwise comparisons using the findMarker function implemented in scran while accounting for the sample effect (**Methods**). The sheet name indicates the cell-type for which marker genes are listed. Genes are ranked based on their top rank across all pairwise comparisons. Top: the minimum rank across all pairwise comparisons. Columns labelled with “logFC.X”: $\log_2$-fold change between the current cell-type and the cell-type X. GeneName: Gene symbol for marker genes. PTM – Peritubular Myoid cells, tMg – testicular Macrophages.

Title: Supplementary Data 4: **Differential expression within each somatic cell-type between P5 and P10.**
Description: Differential gene expression (DE) was tested between pseudo-bulk samples of P5 and P10 for each cell-type using edgeR (**Methods**). Sheets are labelled based on the direction of the DE test. Only genes with FDR < 0.1 are shown. logFC: $\log_2$-fold change between P5 and P10. logCPM: $\log_2$-transformed counts per million (CPM) averaged across P5 and P10. FDR: False discovery rate. GeneName: Gene symbol for marker genes. PTM – Peritubular Myoid cells, tMg – testicular Macrophages.

Title: Supplementary Data 5: **Cluster-specific marker genes for spermatogonia enriched in P5.**
Description: Marker genes were detected across multiple pairwise comparisons using the findMarker function implemented in scran. The sheet name indicates the cell-type for which marker genes are listed. Genes are ranked based on their top rank across all pairwise comparisons. Top: the minimum rank across all pairwise comparisons. Columns labelled with “logFC.X”: $\log_2$-fold change between the current cell-type and the cell-type X. GeneName: Gene symbol

for marker genes. Diff – Differentiated spermatogonia. Undiff – Undifferentiated spermatogonia.

Title: Supplementary Data 6: **Cluster-specific marker genes for spermatogonia enriched in P10 and P15.**
Description: Marker genes were detected across multiple pairwise comparisons using the findMarker function implemented in scran. The sheet name indicates the cell-type for which marker genes are listed. Genes are ranked based on their top rank across all pairwise comparisons. Top: the minimum rank across all pairwise comparisons. Columns labelled with "logFC.X": $\log_2$-fold change between the current cell-type and the cell-type X. GeneName: Gene symbol for marker genes. Diff – Differentiated spermatogonia, Undiff – Undifferentiated spermatogonia, pL – pre-leptotene spermatocytes.

Title: Supplementary Data 7: **Cluster-specific marker genes for EmptyDrops filtered cells at P15.**
Description: Marker genes were detected across multiple pairwise comparisons using the findMarker function implemented in scran. The sheet name indicates the cell-type for which marker genes are listed. Genes are ranked based on their top rank across all pairwise comparisons. Top: the minimum rank across all pairwise comparisons. Columns labelled with "logFC.X": $\log_2$-fold change between the current cell-type and the cell-type X. GeneName: Gene symbol for marker genes.

Title: Supplementary Data 8: **Normalised gene expression correlated to the number of genes expressed in spermatocytes.**
Description: For each gene, its $\log_2$-transformed, normalised expression per cell was correlated with the number of genes expressed. ID: Ensembl gene identifier, rho: Spearman's rho, p.value:

p. value as returned by the correlatedPairs function implemented in scran, FDR: BH-corrected empirical p-value, symbol: Gene symbol.

Title: Supplementary Data 9: **Differential expression within each germ cell-type between Tc1 and Tc0 animals.**
Description: Differential gene expression (DE) was tested between pseudo-bulk samples from Tc1 and Tc0 for each cell-type using edgeR (Methods). A positive $\log_2$-fold change indicates higher expression in Tc1 samples. Human genes can be identified by their "ENSG" ID while mouse genes are labelled with "ENSMUS". Only genes with FDR < 0.1 are shown. logFC: $\log_2$-fold change between Tc1 and Tc0. logCPM: $\log_2$-transformed counts per million (CPM) averaged across Tc1 and Tc0. FDR: False discovery rate. Genename: Gene symbol for marker genes. eP – early-pachytene spermatocytes (SC), mP – mid-pachytene SC, lP – late-pachytene SC, D – Diplotene SC, MI – Metaphase I, MII – Metaphase II, S1-11 – Step 1-11 Spermatids.

Title: Supplementary Data 10: **Normalised gene expression correlated to the number of genes expressed in spermatids.**
Description: For each gene, its $\log_2$-transformed, normalised expression per cell was correlated against the number of genes expressed. ID: Ensembl gene identifier, rho: Spearman's rho, p.value:

p. value as returned by the correlatedPairs function implemented in scran, FDR: BH-corrected empirical p-value, symbol: Gene symbol, group: Geneset identifier (see **Fig. 6B**).

Title: Supplementary Data 11: Categorisation of X-chromosome genes and normalised counts of histone marks in their promoters.
Description: Spermatocytes.K9: Counts per million for H3K9me3 in X-chromosome gene promoters averaged across the two replicates of spermatocytes.

Spermatids.K9: Counts per million for H3K9me3 in X-chromosome gene promoters averaged across the two replicates of spermatids.
avg.K9: Counts per million for H3K9me3 in X-chromosome gene promoters averaged across spermatocytes and spermatids.
Spermatocytes.K4: Counts per million for H3K4me3 in X-chromosome gene promoters averaged across the two replicates of spermatocytes.
Spermatids.K4: Counts per million for H3K4me3 in X-chromosome gene promoters averaged across the two replicates of spermatids.
avg.K4: Counts per million for H3K4me3 in X-chromosome gene promoters averaged across spermatocytes and spermatids.
Spermatocytes.K27: Counts per million for H3K27ac in X-chromosome gene promoters averaged across the two replicates of spermatocytes.
Spermatids.K27: Counts per million for H3K27ac in X-chromosome gene promoters averaged across the two replicates of spermatids.
avg.K27: Counts per million for H3K27ac in X-chromosome gene promoters averaged across spermatocytes and spermatids.
Symbol: Gene symbol
spermatid_specific: Logical variable indicating if the gene is within the set of spermatid specific genes.
Rnf8: Logical variable indicating if this gene is a target of Rnf8[1].
Scml2: Logical variable indicating if this gene is a target of Scml2[1].

Title: Source Data file
Description: Quantitative image analysis of RNAscope® slides was performed on the HALO Image Analysis Platform (Methods). Signal intensity was quantified across annotation layers containing tubules of the same epithelial stage. Total tissue area, total signal count, average signal optical density, signal count per µm^2, and the number of tubules is recorded. Average signal intensity across all tubules of the same epithelial stage is reported in the form of dots (sig

1
