# Supplementary material for: Staged developmental mapping and X chromosome transcriptional dynamics during mouse spermatogenesis
Source: Nat Commun. 2019 Mar 19;10:1251. doi: 10.1038/s41467-019-09182-1 (PMC6424977; doi:10.1038/s41467-019-09182-1)
Supplement: Supplementary file 14 — Reporting Summary [file 41467_2019_9182_MOESM14_ESM.pdf]

nature research

Corresponding author(s): Duncan T Odom and John C Marioni

Last updated by author(s): Jan 25, 2019

Reporting Summary

Nature Research wishes to improve the reproducibility of the work that we publish. This form provides structure for consistency and transparency in reporting. For further information on Nature Research policies, see Authors & Referees and the Editorial Policy Checklist.

Statistics

For all statistical analyses, confirm that the following items are present in the figure legend, table legend, main text, or Methods section.

n/a	Confirmed	
☐	☒	The exact sample size (*n*) for each experimental group/condition, given as a discrete number and unit of measurement
☐	☒	A statement on whether measurements were taken from distinct samples or whether the same sample was measured repeatedly
☐	☒	The statistical test(s) used AND whether they are one- or two-sided<br>*Only common tests should be described solely by name; describe more complex techniques in the Methods section.*
☒	☐	A description of all covariates tested
☐	☒	A description of any assumptions or corrections, such as tests of normality and adjustment for multiple comparisons
☐	☒	A full description of the statistical parameters including central tendency (e.g. means) or other basic estimates (e.g. regression coefficient) AND variation (e.g. standard deviation) or associated estimates of uncertainty (e.g. confidence intervals)
☐	☒	For null hypothesis testing, the test statistic (e.g. *F*, *t*, *r*) with confidence intervals, effect sizes, degrees of freedom and *P* value noted<br>*Give P values as exact values whenever suitable.*
☒	☐	For Bayesian analysis, information on the choice of priors and Markov chain Monte Carlo settings
☒	☐	For hierarchical and complex designs, identification of the appropriate level for tests and full reporting of outcomes
☐	☒	Estimates of effect sizes (e.g. Cohen's *d*, Pearson's *r*), indicating how they were calculated

Our web collection on statistics for biologists contains articles on many of the points above.

Software and code

Policy information about availability of computer code

Data collection	Software to collect FACS data: BD FACSDIVA.
Data analysis	Cell Ranger v1.3.1, STAR v2.5.3, HT-seq v0.9.1, Bowtie2 v.2.3.4.1, R packages: scran, igraph, Rtsne, irlba, DropletUtils, edgeR, princurve, DESeq2, randomForest, csaw, GenomicFeatures.

For manuscripts utilizing custom algorithms or software that are central to the research but not yet described in published literature, software must be made available to editors/reviewers. We strongly encourage code deposition in a community repository (e.g. GitHub). See the Nature Research guidelines for submitting code & software for further information.

Data

Policy information about availability of data

All manuscripts must include a data availability statement. This statement should provide the following information, where applicable:

- Accession codes, unique identifiers, or web links for publicly available datasets
- A list of figures that have associated raw data
- A description of any restrictions on data availability

Data have been deposited in the ArrayExpress database under accession number E-MTAB-6946 for scRNA-Seq data, E-MTAB-6934 for bulk RNA-Seq data and E-MTAB-6932 for CUT&RUN data.

Field-specific reporting

Please select the one below that is the best fit for your research. If you are not sure, read the appropriate sections before making your selection.

☒ Life sciences ☐ Behavioural & social sciences ☐ Ecological, evolutionary & environmental sciences

For a reference copy of the document with all sections, see nature.com/documents/nr-reporting-summary-flat.pdf

Life sciences study design

All studies must disclose on these points even when the disclosure is negative.

Sample size	After passing QC, for adult samples: 3227 cells. P5: 7470 cells, P10: 3010 cells, P15: 4012 cells, P20: 1713 cells, P25: 4184 cells, P30: 2218 cells, P35: 3026 cells, Tc0: 9112 cells, Tc1: 12417 cells, Bulk RNA-seq samples: 30, CUT&RUN sample: 32
Data exclusions	Default CellRanger filtering: We use the CellRanger default threshold to obtain high-quality cells with large numbers of UMIs. We filtered out cells that express less than 1000 genes. Furthermore, we exclude cells with more than 10% of reads mapping to the mitochondrial genome. EmptyDrops filtering: We therefore used emptyDrops function provided in the DropletUtils Bioconductor package to statistically distinguish empty droplets from genuine cells (controlling the FDR to 1%). We filtered out cells with less than 500 genes expressed. Furthermore, we excluded cells with more than 10% or mitochondrial genes expressed.
Replication	Biological replicates (minimum n=2) were performed for adult and P5 single-cell RNA-Seq datasets, the majority of bulk RNA-Seq samples during the juvenile timecourse (except P8, P16, P32, P34 and P35) and for CUT&RUN samples.
Randomization	Not applicable
Blinding	Not applicable

Reporting for specific materials, systems and methods

We require information from authors about some types of materials, experimental systems and methods used in many studies. Here, indicate whether each material, system or method listed is relevant to your study. If you are not sure if a list item applies to your research, read the appropriate section before selecting a response.

Materials & experimental systems

n/a	Involved in the study	
☐	☒	Antibodies
☒	☐	Eukaryotic cell lines
☒	☐	Palaeontology
☐	☒	Animals and other organisms
☒	☐	Human research participants
☒	☐	Clinical data

Methods

n/a	Involved in the study	
☒	☐	ChIP-seq
☐	☒	Flow cytometry
☒	☐	MRI-based neuroimaging

Antibodies

Antibodies used	Anti-Trimethyl Histone H3 (Lys4) Antibody, clone CMA304, 05-1339, Merck-Millipore; Anti-Histone H3 (tri methyl K9) antibody - ChIP Grade, ab8898, Abcam, Anti-Histone H3 (acetyl K27) antibody - ChIP Grade, ab4729, Abcam, Anti-phospho-Histone H3 (ser10) Antibody, Mitosis Marker.
Validation	Antibodies were validated by the company

Animals and other organisms

Policy information about studies involving animals; ARRIVE guidelines recommended for reporting animal research

Laboratory animals	C57BL/6J mice were obtained from Charles Rivers Laboratories and maintained using standard husbandry procedures. Male mice at 9-weeks of age were used for adult timepoints and juvenile mice were sacrificed at various postnatal (P) timepoints including P5, P6, P8, P10, P12, P14, P15, P16, P18, P20, P22, P24, P25, P26, P28, P30, P32, P34 and P35.<br>The Tc1 trans-chromosomic mouse line was originally obtained from Dr. E. Fisher and Dr. V. Tybulewizc (O'Doherty 2005 Science) and is maintained by breeding Tc1-positive females to male (129S8 x C57BL/6J) F1 mice. Male Tc1 mice and wild-type littermate controls (Tc0) have been used at 8-9 weeks of age.
Wild animals	The study did not involve wild animals

Field-collected samples	The study did not involve wild animals
Ethics oversight	This investigation was approved by the Animal Welfare and Ethics Review Board and followed the Cambridge Institute guidelines for the use of animals in experimental studies under Home Office licences PPL 70/7535 until February 2018 and PPL P9855D13B from March 2018. All animal experimentation was carried out in accordance with the Animals (Scientific Procedures) Act 1986 (United Kingdom) and conformed to the Animal Research: Reporting of In Vivo Experiments (ARRIVE) guidelines developed by the National Centre for the Replacement, Refinement and Reduction of Animals in research (NC3Rs).

Note that full information on the approval of the study protocol must also be provided in the manuscript.

Flow Cytometry

Plots

Confirm that:

☒ The axis labels state the marker and fluorochrome used (e.g. CD4-FITC).

☒ The axis scales are clearly visible. Include numbers along axes only for bottom left plot of group (a 'group' is an analysis of identical markers).

☒ All plots are contour plots with outliers or pseudocolor plots.

☒ A numerical value for number of cells or percentage (with statistics) is provided.

Methodology

Sample preparation	Murine testes were dissociated as previously described (Ernst et al. 2016 eLife) and stained with Hoechst 33342 (H3570, ThermoFisher) for 45 minutes at 37C.
Instrument	Aria IIu, Becton Dickinson
Software	BD FACSDiva 8.0.1
Cell population abundance	Spermatocytes made up approximately 14% of the cell population and spermatids ranged between 15-26% of the cell population.
Gating strategy	Dead cell exclusion was performed by gating on PI versus FSC and PI versus SSC, followed by doublet exclusion gating on Hoechst Blue versus Hoechst Blue. Cell populations of interest were identified based on DNA content (4N spermatocytes and 1N spermatids) as previously described (Bastos et al. 2005)

☒ Tick this box to confirm that a figure exemplifying the gating strategy is provided in the Supplementary Information.
